# MT-GCNN: Multi-Task Learning with Gated Convolution for Multiple Transmitters Localization in Urban Scenarios

**DOI:** 10.3390/s22228674

**Published:** 2022-11-10

**Authors:** Wenyu Wang, Lei Zhu, Zhen Huang, Baozhu Li, Lu Yu, Kaixin Cheng

**Affiliations:** 1 College of Communications Engineering, Army Engineering University of PLA, Nanjing 210001, China; 2 Beijing National Research Center for Information Science and Technology, Tsinghua University, Beijing 100089, China

**Keywords:** multiple transmitters localization, deep learning, multi-task learning, nonline-of-sight propagation, sparse sensors

## Abstract

With the advance of the Internet of things (IoT), localization is essential in varied services. In urban scenarios, multiple transmitters localization is faced with challenges such as nonline-of-sight (NLOS) propagation and limited deployment of sensors. To this end, this paper proposes the MT-GCNN (Multi-Task Gated Convolutional Neural Network), a novel multiple transmitters localization scheme based on deep multi-task learning, to learn the NLOS propagation features and achieve the localization. The multi-task learning network decomposes the problem into a coarse localization task and a fine correction task. In particular, the MT-GCNN uses an improved gated convolution module to extract features from sparse sensing data more effectively. In the training stage, a joint loss function is proposed to optimize the two branches of tasks. In the testing stage, the well-trained MT-GCNN model predicts the classified grids and corresponding biases jointly to improve the overall performance of localization. In the urban scenarios challenged by NLOS propagation and sparse deployment of sensors, numerical simulations demonstrate that the proposed MT-GCNN framework has more accurate and robust performance than other algorithms.

## 1. Introduction

With the prevalence of wireless devices and the application of wireless sensor networks (WSNs), the localization of transmitters has attracted extensive attention in the Internet of things (IoT) era. Localization is important in commercial, industrial, and defense tasks, such as activity monitoring, spectrum cognition, and target tracking in urban scenarios. Generally, the measurements of sensors in WSNs are collected and processed for localization in the form of time of arrival (TOA), time difference of arrival (TDOA), angle of arrival (AOA), or received signal strength (RSS). Many valuable studies based on the above measurements have focused on the single transmitter localization [1,2,3,4]. Notably, due to the complex electromagnetic propagation conditions in urban scenarios, multiple transmitters localization, a quite challenging issue, has been increasingly researched.

Some existing multiple transmitters localization approaches estimate the position of each transmitter separately, depending on the distinguishability of signals [5,6]. Nonetheless, signals from different transmitters are intractable to separate, especially in non-cooperative and spectrum-shared situations. Under conditions where only superimposed measurements are accessible in WSNs, traditional range-based algorithms are unsuitable for multiple transmitters localization. Without the need for signal separation, compressive sensing (CS) methods have been utilized to transform the localization problem into a sparse reconstruction problem in plenty of research [7,8]. The transmitters and sensors are assumed to be located at the fixed grid center. Jiang et al. [7] presented a robust CS-based localization scheme with the aid of Laplace prior, but in practice, the off-grid problem of transmitters can cause larger positioning errors. A grid evolution algorithm was proposed to establish a more accurate sparse recovery framework for off-grid targets localization in [8]. In addition, numerous algorithms were developed to improve the CS-based localization [9,10].

Although widely applied for multiple transmitters localization, CS-based methods implicitly assume an experiential or statistical electromagnetic wave propagation model. In urban scenarios, nonline-of-sight (NLOS) propagation is unavoidable due to the dense distribution of buildings, and results in extra errors for traditional propagation calculation. Therefore, the experiential and statistical propagation models fail to effectively fit the urban NLOS propagation [11,12].

Thanks to the marvelous development of artificial intelligence, deep learning (DL) is proved effective in learning complex propagation features and improving localization performance [13,14,15]. In the International Conference on Indoor Positioning and Indoor Navigation (IPIN) Competition, which is highly relevant for the indoor positioning community, many teams successfully profited from deep learning techniques and see a prospect of more widespread adoption in indoor positioning in the near future [16,17]. One of the contributions of the Naver Labs Europe (NLE) Team is that the magnetic sensing data were encoded into 2D images, and then the deep neural networks were applied to capture the hidden correlations in the inputs and predict the positions. Team KawaguchiLab utilized Long Short-Term Memory (LSTM) to achieve the speed estimation of dead reckoning and gained robustness to noisy data. The SZU-Mellivora Capensis team proposed a recurrent neural network (RNN) framework to update the target’s position. In addition, Team Yai trained six deep neural network (DNN) based classifiers and combined them to obtain a more precise localization result. Other research teams are also interested in the deep learning based localization. Designed for single-target localization, CellinDeep [18] divided the study area into grids and adopted a classification DNN to predict the probability of each grid. Then, the spatially weighted average was used to solve the off-grid problem in localization. Practical experiments of CellinDeep in complex environments validated its effectiveness in recognizing propagation features. In addition, some fingerprint-based localization methods, which consist of fingerprint database construction and fingerprint matching [19], also apply the DL framework to train fingerprints instead of storing all the data. The wavefingerprints can be transformed into feature-based fingerprints to reflect the propagation features, and the neural networks are capable of mapping the fingerprints with the location features [20]. Essentially, the function of the neural networks among the DL-based localization techniques is to learn the propagation features and look for the mapping between the sensing data and the locations. In addition, driven by data, the DL-based localization usually contains two phases as well, i.e., offline training phase and online testing phase.

Regarding multiple transmitters localization, DL is still effective to capture the propagation features and achieve the localization with no need for signal separation. DeepTxFinder [21] predicted the number of transmitters at first and then regressed the coordinates directly. HMTLNet [22] aimed to transform the localization into a heatmap regression problem. DeepMTL [23] solved the problem with convolutional neural network (CNN) models from the view of computer vision. However, the regression-based or image-based schemes require hundreds of sensors [24], and these methods have to predict the continuous location variables in the solution space. Under the NLOS propagation and sparse sensing conditions, it is intractable to achieve the comprehensive perception of the mapping between the sensing data and the location variables [25].

To lower the difficulty of regressive mapping, this paper utilizes the classification to narrow the solution space by dividing the continuous study area into grids at first. Regarding the coordinates of grid centers as the coarse localization results, the discrete classification leads to off-grid errors. To gain continuous and more accurate results, the regression of the biases off the grid centers is expected to correct the localization. Therefore, this paper proposes a multi-task gated convolutional neural network (MT-GCNN) to decompose the multiple transmitters localization problem into a coarse localization task and a fine correction task. The two tasks in the proposed MT-GCNN complement each other. More importantly, an improved gated convolution module is proposed as the backbone of MT-GCNN to enhance the feature capture capacity. The fusion of features in the improved gated convolution module and the parallel processing in the multi-task learning framework facilitate a more accurate performance of multiple transmitters localization.

The main contributions in this paper can be summarised as follows:

1. In the proposed MT-GCNN, the multi-label classification and the bias regression are combined to predict the coarse results and correct biases. A joint loss function is also designed to train the two tasks simultaneously.
2. Considering the challenges of NLOS propagation and limited layouts of sensors, an improved gated convolution module is applied for feature extraction in MT-GCNN. The gated mechanism [26] and convolutional module are combined to fuse the multi-dimensional features of sparse sensing data in complex environments.
3. With the aid of the simulation software Winprop, the proposed localization scheme is validated based on the urban NLOS propagation datasets. Moreover, this paper analyzes the localization performance of different factors, including the number of transmitters, the number of sensors, the impact of measurement noise, and the complexity of models.

The following contents are organized as follows: Section 2 presents the multi-task localization framework’s problem formulation and data preparation, Section 3 introduces the details of the proposed MT-GCNN model for multiple transmitters localization, and the simulation results and performance evaluation are shown in Section 4. Finally, conclusions are included in Section 5.

## 2. Problem Formulation

We suppose there are *M* transmitters and *N* sensors in a two-dimensional study area, where the unknown position of the *i*th transmitter is denoted as $L_{T,i}=\left(x_{i},y_{i}\right),i=1,2,\ldots ,M$, and each sensor is given by the known coordinate $L_{S,i}=\left(x_{i},y_{i}\right),i=1,2,\ldots ,N$. According to the function of the deployed sensors, the superimposed signal measurements from transmitters, such as RSS, TOA, TDOA, and AOA. RSS is easily accessible and is thus chosen as the study measurements of sensors. Focusing on the sparse layouts of sensors, we denote the RSS measurements from all sensors as a vector $R=(r_{1},r_{2},\ldots ,r_{N})$, a sample of data-driven neural network input. Notably, the sensing data are utilized to learn the propagation characteristics in the study area. Therefore, the proposed localization scheme is not limited to the type of RSS measurements and requires no propagation model assumption.

Multi-task learning (MTL) has been increasingly used to improve the overall performance in many fields [27,28]. Generally, multiple tasks are conducted in a unified network simultaneously and share in the representation of features in the bottom layers. In this paper, the MTL framework is utilized to achieve the following coarse localization task and fine correction task.

The typical multi-classification algorithms inspire the coarse localization branch for single transmitter localization [18,29,30]. Based on the fact that the propagation features are similar in a small block, the continuous study area is divided into *K* grids with the length of *a* m and the width of *b* m. Each grid represents a category for the classifier. The mentioned classification-based localization algorithms categorize the single transmitter into a fixed grid, but they are unsuitable for multiple transmitter localization. In this paper, the multi-classification is generalized into the multi-label classification to solve the coarse-grained multiple transmitters localization. Assuming that transmitters are located in different grids, this branch aims to determine which *M* categories (i.e., grids) correspond with the RSS vector. Thus, the classification label $Y_{cla}$ of the training dataset is set as a 0–1 vector with the length of *K*, which means the transmitters are located in the *M* grids with the value 1.

Unavoidably, the coarse classification leads to off-grid errors, and the achievable precision is limited to the discrete grids. The fine correction branch is designed to predict the biases off the grids to gain continuous localization results. The bias label is expressed as $Y_{bias}$ with the size of 2×K. In $Y_{bias}$, the first row contains the x-coordinate biases off the centers of grids marked with 1 in $Y_{cla}$, whereas the second row denotes y-coordinate biases. The $Y_{bias}$ values corresponding to those grids marked with 0 in $Y_{cla}$ are set to zeros. Finally, the classification label $Y_{cla}$ and the bias label $Y_{bias}$ are stacked as

$$Y_{3\times K}=\left(\begin{array}{c} Y_{cla,1\times K}\\ Y_{bias,2\times K} \end{array}\right).$$

**Figure 1:**
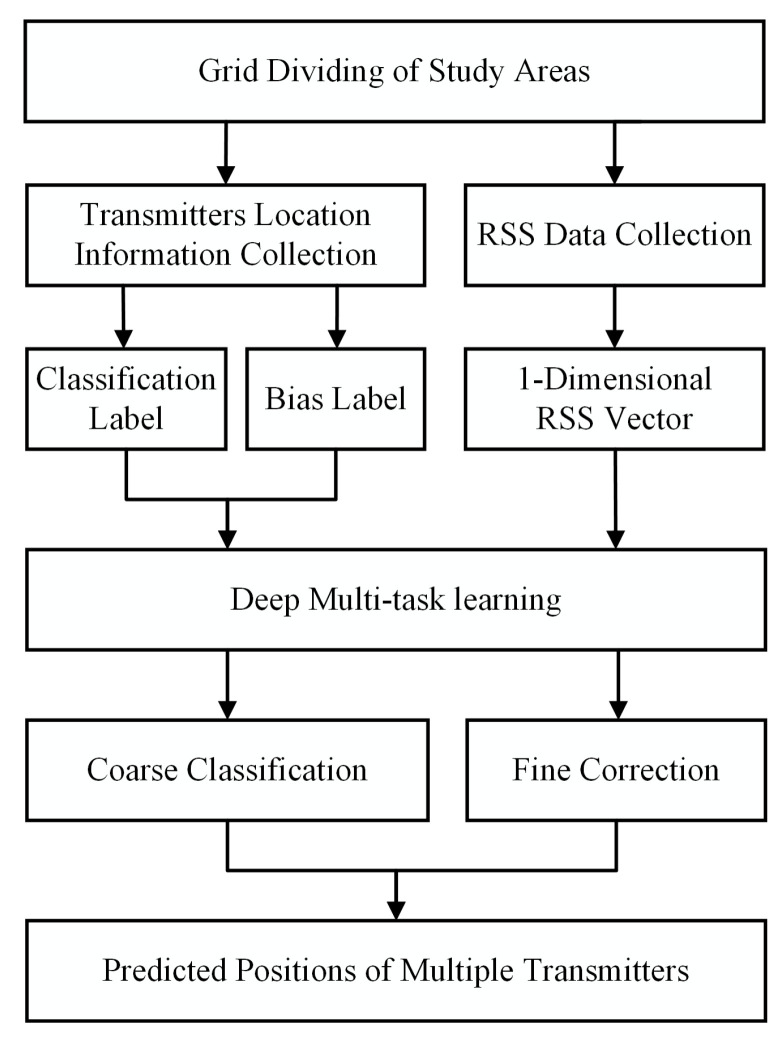
The localization scheme based on the deep multi-task learning.

The localization scheme based on deep multi-task learning is shown in Figure 1. Firstly, the position of each transmitter and the aggregated RSS data are collected after dividing the study area into grids. Next, the RSS data are processed into a vector of length as the inputs. Then, the transmitters are categorized into grids, and the biases off the centers are calculated to generate the classification and bias labels. Each dataset sample prepared for the DL-based localization scheme consists of the RSS input vector and the stacked label. In the training stage, transmitters are randomly deployed to collect sufficient samples. In the testing stage, the well-trained MT-GCNN model jointly predicts the classified grids and the corresponding biases. The final localization results are calculated by the sum of the coarse results and the biases, whereas labels are only used to evaluate the performance of the proposed localization scheme.

## 3. MT-GCNN Model for Multiple Transmitters Localization

This section presents the detailed implementation and configuration of the proposed MT-GCNN model. Firstly, we explain the design principle of core modules, including the convolution module, the gated mechanism, and the multi-task module. Next, the joint loss function and the localization procedure are illustrated.

### 3.1. The Design of MT-GCNN

**Figure 2:**
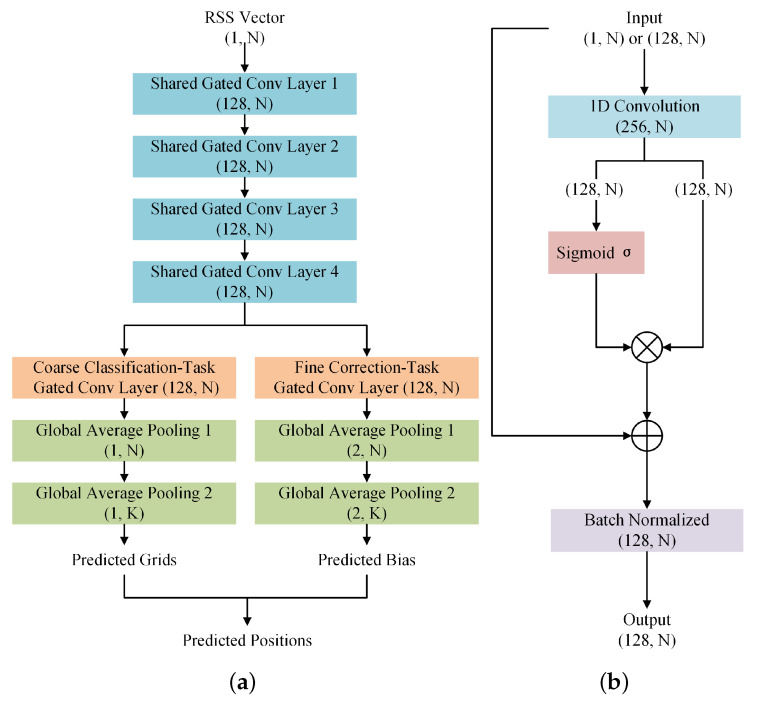
The architecture of (**a**) the MT-GCNN framework and (**b**) the improved gated convolution module.

As shown in Figure 2, MT-GCNN is a multi-task learning framework with the backbone of an improved gated convolution module. In many fields [31,32], the gated mechanism empowers the features to flow through different levels, achieving the fusion of linear and nonlinear features transformation. Considering the limited information in the inputs when the sensors are deployed sparsely, it is more difficult to learn the hidden NLOS propagation features, and the performance of localization gets worse. In this section, an improved gated convolution module is designed for sparse sensing data, resulting in a richer capture and fusion for multi-dimensional features.

Specifically, the 1D convolutional layer with 256 channels and 5 × 5-sized kennels is used to extract local features from the input $R=(r_{1},r_{2},\ldots ,r_{N})$. Proper padding is applied to keep the same length of feature maps. Generally, common activation functions, such as ReLu, Sigmoid, and Tanh, are set to introduce the nonlinear relationships into the features. Fewer original propagation features hidden in the inputs can be observed as the number of sensors reduces. Thus, the output feature maps are possibly erroneous. An improved gated unit is incorporated into the convolution module to perceive features under the sparse sensing condition fully. Specifically, the feature maps with the size of (256, *N*) are evenly divided into a linear path and a nonlinear path. The former is used to keep the standard convolutional features, which contributes to alleviating the vanishing or exploding gradient problem. The latter retains the nonlinear capabilities via Sigmoid activation function. The output formula of this module is expressed as [26]:

(1) $$O_{GC}={\mathrm{Conv}1D}_{1}\left(I\right)⊗\sigma \left({\mathrm{Conv}1D}_{2}\left(I\right)\right).$$

Herein, I is the input of the gated convolution module, ⊗ means the element-wise multiplication, and σ represents the Sigmoid activation function, constraining the value of the nonlinear branch to be within [0, 1]. In addition, the output size $O_{GC}$ is changed into (128, *N*) after the multiplication.

In particular, a skip connection of the residual structure is added to the 1D gated convolution module to enhance the fusion of multi-dimensional features. The improved output formula is as follows:

(2) $$O_{GC}=I+{\mathrm{Conv}1D}_{1}\left(I\right)⊗\sigma \left({\mathrm{Conv}1D}_{2}\left(I\right)\right).$$

From the view of information flow, the rewritten equation is derived as follows:

(3) $$\begin{array}{cc} O_{GC} & =I+\left({\mathrm{Conv}1D}_{1}\left(I\right)-I\right)⊗\sigma \left({\mathrm{Conv}1D}_{2}\left(I\right)\right)\\ \; & =I⊗\left[1-\sigma \left({\mathrm{Conv}1D}_{2}\left(I\right)\right)\right]+{\mathrm{Conv}1D}_{1}\left(I\right)⊗\sigma \left({\mathrm{Conv}1D}_{2}\left(I\right)\right)\\ \; & =I⊗\left(1-\sigma \right)+{\mathrm{Conv}1D}_{1}\left(I\right)⊗\sigma , \end{array}$$

where $\sigma =\sigma \left({\mathrm{Conv}1D}_{2}\left(I\right)\right)$. Due to the linear transformation of convolution, ${\mathrm{Conv}1D}_{1}\left(I\right)-I$ is equal to the ${\mathrm{Conv}1D}_{1}\left(I\right)$ in Equation (2). Therefore, the information of features passes directly with the probability of 1−σ, while flowing through layers with the probability of σ after transformation. Integrating original, convolutional, and activated nonlinear features, the improved gated convolution module ensures that the features flow through different channels before they are fused.

In addition, batch normalization, a technique to train deep networks, is utilized for feature scaling [33], accelerating the convergence and making it easier for the network to learn the fusion features. Eventually, based on Equation (3), the final output of the improved gated convolution module is updated as:

(4) $$O_{GC}=\mathrm{BN}\left(O_{GC}\right).$$

In the proposed MT-GCNN model, four improved gated convolutional layers are stacked to extract shared feature maps with the size of (128, *N*) based on the input $R=(r_{1},r_{2},\ldots ,r_{N})$. Then, a multi-label classification branch and a bias regression branch are used to predict the located grids and the biases off the centers. Both consist of an improved gated convolutional layer and two global average pooling layers. In the classifier, the first pooling layer is applied to unify the 128 channels into 1, and the other layer transforms the length of *N* into *K* to generate the output $O_{cla}$ with the size of (1, *K*). Elements in $O_{cla}$ represent the scores of each class calculated by the network. In the regression branch, the number of channels is changed into 2 to generate the (2, *K*) sized output $O_{bias}$ through the global average pooling layers.

Consequently, the improved gated convolution module is expected to largely affect the extraction and fusion of multi-dimensional features. Moreover, two output branches of the MT-GCNN model are designed to conduct coarse localization and fine correction for multiple transmitters localization.

### 3.2. Joint Loss Function

Design and minimization of loss functions are necessary to train deep networks. In our MT-GCNN model, the joint loss function is set as the combination of each task’s loss, which is explained in detail as follows.

**(1) Multi-label Classification Loss:** For a traditional classifier, the cross-entropy function is generally combined with the Softmax function to predict the class, which can be expressed as:

(5) $$L_{cross-entropy}=-\log\frac{e^{s_{t}}}{\sum_{i=1}^{K}e^{s_{i}}}=\log\left(1+\sum_{i=1,i\neq t}^{K}e^{s_{i}-s_{t}}\right),$$

where $s_{i}$ represents the score of the *i*th class (with a total of *K* classes), and the *t*th class is referred to as the target class. Regarding the multiple transmitters localization as multi-label classification, we can achieve *M* target and K−M non-target classes. Inspired by a unified loss function proposed in [34], the target classes and the non-target classes can be analogized as the intra-class similarity $s_{p}$ and the inter-class similarity $s_{n}$ in Equation (6):

(6) $$L_{uni}=\log\left(1+\sum_{j}e^{\gamma (s_{n}^{j}+m)}\sum_{i}e^{\gamma (-s_{p}^{i})}\right),$$

where γ and *m* are the scale factor and the interval for better similarity separation, respectively.

Similarly, a loss function for the multi-label classification task is extended as follows to maximize the scores of target classes and minimize the scores of non-target classes:

(7) $$L_{cla}=\log\left(1+\sum_{j\in \Omega_{N}}e^{s_{j}}\sum_{i\in \Omega_{T}}e^{-s_{i}}\right).$$

Here, the set $\Omega_{T}$ contains *M* target classes, and the set $\Omega_{N}$ has K−M non-target classes. Equation (5) is a special form of Equation (7).

**(2) Masked Regression Loss:** The output $O_{bias}$ predicts the biases off grids. A mean squared error (MSE) function is applied to calculate the loss between the predicted output $O_{bias}$ and the bias label $Y_{bias}$. In addition, the 0-1 classification label $Y_{cla}$ is regarded as a mask to concentrate on optimizing the bias prediction of target grids. Thus, the masked regression loss for bias prediction in the fine correction task is expressed as:

(8) $$L_{bias}=\frac{1}{N_{s}}\sum_{i=1}^{N_{s}}{∥Y_{cla}⊗\left(O_{bias}-Y_{bias}\right)∥}^{2},$$

where $N_{s}$ is the number of samples, ⊗ represents the element-wise multiplication, and ${∥\cdot ∥}^{2}$ calculates the sum of squares.

Therefore, the joint loss function is expressed as the loss sum of each task:

(9) $$L_{joint}=L_{cla}+L_{bias}.$$

### 3.3. Training and Localization

In the training stage, the backpropagation algorithm and the adaptive moment estimation (Adam) optimizer are used to calculate gradients and minimize the joint loss to train the MT-GCNN model end-to-end with an initial learning rate of 0.01. The exponential decay rates of first-order and second-order moment estimation are 0.5 and 0.9, respectively. The Adam optimizer is self-adaptive to tune the learning rate with the parameters update.

During the online localization, the trained MT-GCNN model depends only on the sensing data $R=(r_{1},r_{2},\ldots ,r_{N})$ from sensors to predict the positions of multiple transmitters. The output of the coarse localization branch is $O_{cla}=\left(s_{1},s_{2},\ldots ,s_{K}\right)$, and the Softmax function is employed to convert the scores into the joint probability of each grid:

(10) $$P\left(s_{i}\right)=\frac{e^{s_{i}}}{\sum_{j=1}^{K}e^{s_{j}}}.$$

The joint probability reflects grids where the transmitters are possibly located. The centers of those grids with maximum *M* probability are selected as the coarse localization results. It can be assumed that the set $G=\{g_{1},g_{2},\ldots ,g_{M}\}$ contains the indexes of the selected grids and $L_{c,i}=\left(x_{c,i},y_{c,i}\right),\;i=1,2,\ldots ,K$ represents the center coordinate of the grid *i*.

Meanwhile, the output

$$O_{bias}=\left(\begin{array}{c} a_{1},a_{2},\ldots ,a_{K}\\ b_{1},b_{2},\ldots ,b_{K} \end{array}\right)$$

is predicted as the biases of the grids to achieve continuous and accurate localization through the fine correction branch. The final localization result $L_{i}^{*}=\left(x_{i}^{*},y_{i}^{*}\right),i=1,2,\ldots ,M$ is calculated as follows:

(11) $$\begin{array}{cc} x_{i}^{*} & =x_{c,g_{i}}+a_{g_{i}},\\ y_{i}^{*} & =y_{c,g_{i}}+b_{g_{i}}. \end{array}$$

## 4. Numerical Evaluation

In this section, extensive simulations are conducted to evaluate the effectiveness and robustness of the proposed MT-GCNN multiple transmitters localization framework. Firstly, a more precise propagation calculation is used to generate simulated datasets with the assistance of the Winprop software. Next, the performance of localization under different conditions is discussed and compared.

### 4.1. Simulation Setup

**Figure 3:**
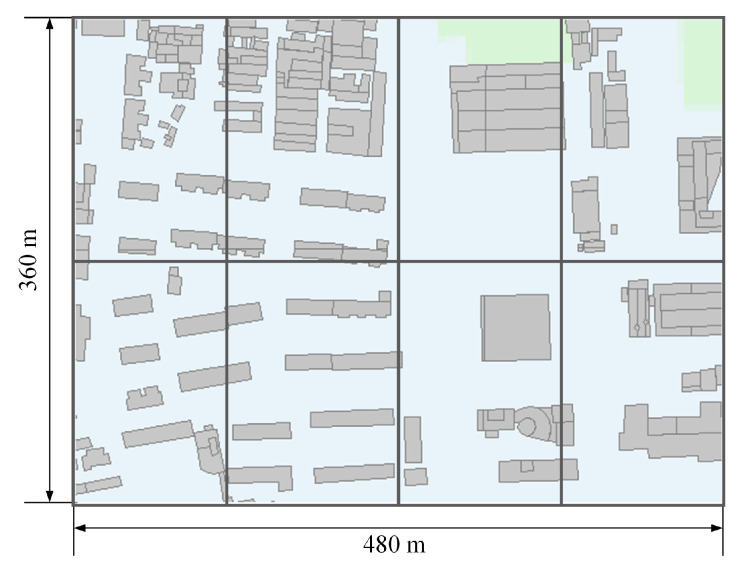
The gridding of the study area in Tsinghua University.

Winprop [35] is a simulation software offering precise electromagnetic wave propagation calculation based on the dominant path model (DPM) [36] and 3D building map. This paper uses the Winprop software to simulate the actual urban electromagnetic wave propagation and generate corresponding data to verify the proposed framework. As is shown in Figure 3, a 480 m × 360 m-sized study area in Tsinghua University is selected to construct the actual geographic model and conduct propagation simulations. The default size of dividing grids is 120 m × 180 m, and the number of grids is K=8. In this study area, the default number of transmitters is set as M=3. We assume that *M* transmitters are randomly located in different grids with the same frequency of 1800 MHz and the same transmitted power of 43 dBm. *N* sensors are uniformly deployed in the study area to receive the RSS values from transmitters, with the maximum value being set into 12. In the training datasets, 19,200 samples are collected to train the proposed MT-GCNN, and 2400 samples are prepared for validation and testing datasets to evaluate the localization performance. The training epoch is 100, and other network parameters have been set as the detailed configuration in Section 3. All numerical simulations are conducted on an RTX2080ti GPU with 96 GB of RAM, and PyTorch is used as the DL algorithm basis.

### 4.2. Performance Comparison

To verify the effectiveness and robustness of the proposed MT-GCNN framework, we adopt two existing methods and two classification-based network structures for comparison, including DeepMTL [23] for multiple transmitters localization, CellinDeep [18] for single transmitter localization, MLP [30] structure with full connection layers, and GCNN structure only with the improved gated convolution. Since the DeepMTL relies on the dense deployment of sensors, it fails in convergence to achieve multiple transmitters localization under the sparse sensing condition of fewer than 12 sensors. In addition, another regression-based DeepTxFinder [21] is incapable of localization on the same urban datasets. CellinDeep applies the classification and the spatially weighted average. MLP and GCNN structures are used for the coarse classification-based localization without the multi-task learning framework.

There are two performance indicators for comparing localization algorithms in this paper. One is the grid-classification accuracy:

(12) $$\alpha =\frac{n_{t}}{N_{s}\cdot M}\times 100\%,$$

which shows the performance of the coarse localization task. $n_{t}$ is the number of the correct classification for transmitters, and $N_{s}\cdot M$ refers to the total number of transmitters in all samples. The other indicator is the mean positioning error to evaluate the final localization results:

(13) $$\varepsilon =\frac{1}{N_{s}\cdot M}\sum_{i=1}^{N_{s}\cdot M}\sqrt{{\left(x_{i}^{*}-x_{i}\right)}^{2}+{\left(y_{i}^{*}-y_{i}\right)}^{2}}.$$

The proposed MT-GCNN is compared with the algorithms from different transmitters, sensors, and measurement noise conditions, and the complexity of models is also analyzed. The results of the comparative experiments are as below:

**(1) With a different amount of transmitters:** The proposed MT-GCNN framework is also suitable for single transmitter localization. Firstly, we test the performance of single transmitter localization of N=4 sensors. Table 1 compares grid-classification accuracy and the mean positioning error with different algorithms. Compared with those regression-based methods, the four classification-based algorithms are easier to converge. Limited by the size of grids, the classification-based structures such as GCNN and MLP suffer from the off-grid problem, and the spatially weighted average in CellinDeep is ineffective in improving the localization. In contrast, the parallel processing of coarse classification task and fine regression task in MT-GCNN is effective to improve the localization. The grid-classification accuracy of MT-GCNN achieves 98.25%, and the mean positioning error is significantly reduced by 90.45% from about 60 m to 5.73 m. In addition, the total number of sensors is N=4, which is an extremely sparse sensing condition.

**Figure 4:**
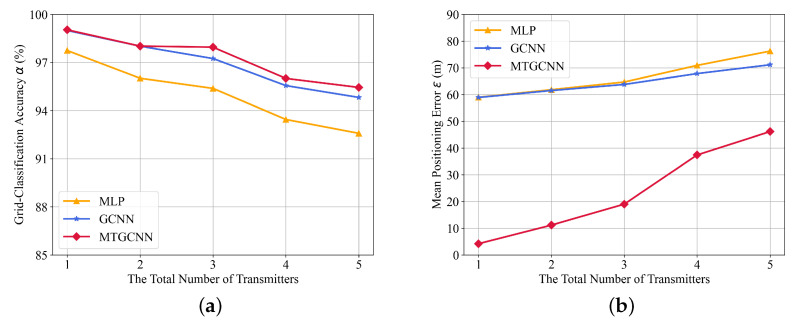
The localization results with a different number of transmitters. (**a**) grid-classification accuracy and (**b**) mean positioning error.

Furthermore, Figure 4 illustrates the performance as the number of transmitters varies from 1 to 5 when the total number of sensors is N=12. Generally, the localization performance degrades due to the increase of transmitters because more propagation information is necessary for DL-based networks to detect and localize more transmitters. Nevertheless, the proposed MT-GCNN achieves the best performance with the fusion of features and the multi-task framework.

**Figure 5:**
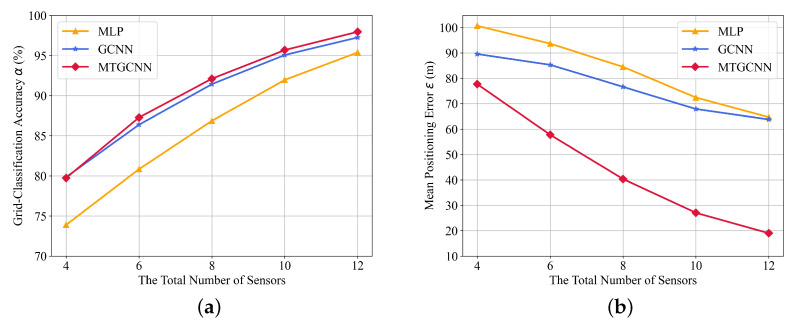
The localization results with a different number of sensors. (**a**) grid-classification accuracy and (**b**) mean positioning error.

**(2) With a different number of sensors: **Figure 5 shows the localization results with different amounts of sensors when the total transmitters are M=3. As the amount of sensors reduces, the grid-classification accuracy decreases, and the mean positioning error increases. In terms of grid-classification accuracy in Figure 5a, the improved gated convolution module in MT-GCNN and GCNN is more effective in extracting NLOS propagation features than full connection layers in the traditional MLP structure. The proposed multi-task framework is slightly improved compared with GCNN in the coarse localization task. Regarding the mean positioning error in Figure 5b, the bias prediction of the fine correction task in MT-GCNN can improve the final localization performance under sparse layouts of less than 12 sensors.

**Figure 6:**
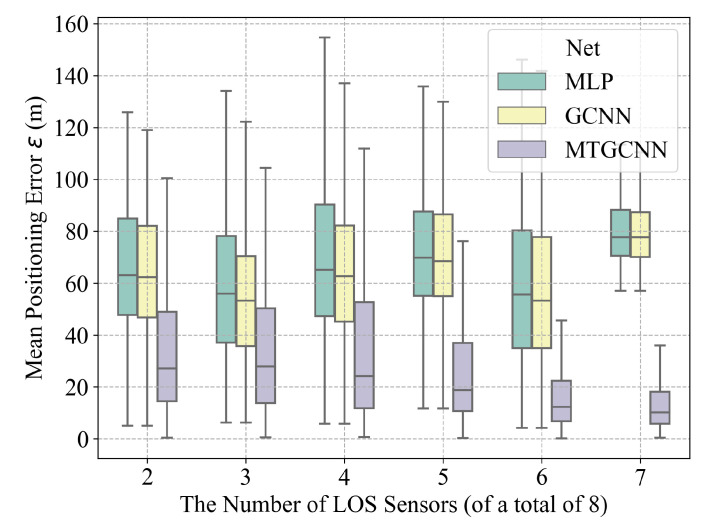
The localization results with a different number of LOS sensors (a total of eight sensors).

With N=8 sensors, Figure 6 presents a box-plot for the localization results as the number of LOS sensors varies from 2 to 7. Compared with the MLP and GCNN structure, MT-GCNN has the lowest median localization error of below 30 m and has superior interquartile range performance, especially with more LOS sensors. The proposed MT-GCNN outperforms the MLP and the GCNN despite the number of LOS sensors, suggesting the effectiveness and robustness of the proposed localization framework under the NLOS propagation.

**Figure 7:**
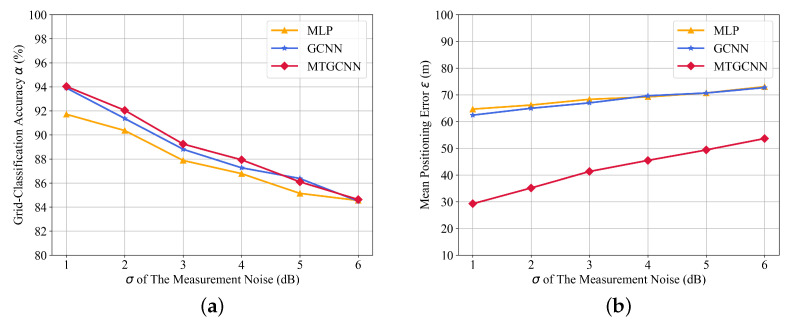
The localization results with different σ (dB) of noise. (**a**) grid-classification accuracy and (**b**) mean positioning error.

**(3) The impact of noise:** Inevitably, the measurements of sensors are prone to external noise. Gaussian white noise $N(0,\sigma^{2})$ is added to the testing data to evaluate the robustness of the proposed MT-GCNN, where σ (dB) is the standard deviation. With the fixed number of transmitters as M=3 and a total number of sensors as N=12, Figure 7 shows the impact of noise on the localization performance and the comparative results of MT-GCNN, GCNN, and MLP. As the standard deviation σ of noise varies from 1 to 6 (dB), the grid classification accuracy decreases from 94% to 85% and the localization error of MT-GCNN increases from 30 m to 50 m. However, the proposed MT-GCNN still achieves the superior performance than GCNN and MLP under different noise conditions.

**(4) The complexity of models:** Mathematical complexity of models usually contains the time and space complexity. Specifically, the time complexity reflects the Floating Point Operations (FLOPs) of models, while space complexity represents the total amount of parameters in layers and operational process. In the MLP, GCNN and MT-GCNN, the 1D convolutional layers, the element-wise multiplication in the gated mechanism, the global average pooling layers and the full connection layers are mainly considered to calculate the complexity mathematically. For the 1D convolutional layers, the time and space complexity are shown as Equations (14) and (15):

(14) $$\mathrm{Time}∼O\left(\sum_{i=1}^{N_{c}}l_{i}\cdot k_{i}\cdot C_{i-1}\cdot C_{i}\right),$$

(15) $$\mathrm{Space}∼O\left(\sum_{i=1}^{N_{c}}k_{i}\cdot C_{i-1}\cdot C_{i}+\sum_{i=1}k_{i}\cdot C_{i}\right),$$

where $N_{c}$ represents the total amount of the convolutional layers in the networks. $N_{c}=6$ in MT-GCNN, while $N_{c}=5$ in GCNN. *i* represents the *i*th layer, $l_{i}$ is the length of the inputs, and $k_{i}$ is the size of the kennels. *C* is the number of channels.

For the gated mechanism, the time and space complexity depend on the element-wise multiplication:

(16) $$\mathrm{Time}∼O\left(\sum_{i=1}^{N_{c}}l_{i}\cdot C_{i}\right),$$

(17) $$\mathrm{Space}∼O\left(\sum_{i=1}^{N_{c}}l_{i}\cdot C_{i}\right).$$

For the global average pooling layers, the time and space complexity depend on the number of channels:

(18) $$\mathrm{Time}∼O\left(\sum_{i=1}^{N_{p}}C_{i-1}\cdot C_{i}\right),$$

(19) $$\mathrm{Space}∼O\left(\sum_{i=1}^{N_{p}}C_{i-1}\cdot C_{i}\right).$$

where $N_{p}$ represents the total amount of the global average pooling layers in the networks. $N_{p}=4$ in MT-GCNN, while $N_{p}=2$ in GCNN.

For the full connection layers, the time and space complexity are shown as Equations (20) and (21):

(20) $$\mathrm{Time}∼O\left(\sum_{i=1}^{N_{f}}D_{i-1}\cdot D_{i}\right),$$

(21) $$\mathrm{Space}∼O\left(\sum_{i=1}^{N_{f}}D_{i-1}\cdot D_{i}\right),$$

where $N_{f}$ is the total amount of the full connection layers in the networks. In MLP, $N_{f}=4$. *D* is the flatten size of the features.

In addition, from the view of the experimental analysis, testing time reflects the time efficiency, which is also calculated to compare MLP, GCNN, and the proposed MT-GCNN.

With the number of sensors N=12 and the number of grids K=8, Table 2 presents the comparison of the mathematical complexity and testing time of MLP, GCNN, and the proposed MT-GCNN. The results show that the gated convolution in GCNN, and MT-GCNN results in higher complexity than MLP. However, the actual testing time is only slightly different. It is attractive to yield an improvement in localization accuracy with acceptable complexity.

## 5. Conclusions

This paper proposes the MT-GCNN model, a novel multiple transmitters localization scheme based on deep multi-task learning. The problem of multiple transmitters localization in the scheme is transformed into a coarse multi-label localization task and a fine bias correction task. Accordingly, the multi-label localization loss and the masked regression loss are combined. The proposed MT-GCNN is trained end to end by minimizing the joint loss function. Furthermore, an improved gated convolution module is applied in the MT-GCNN framework to enhance the capture of NLOS propagation features of the sparse sensing data. With the reliable simulations in Winprop, the numerical results validate the effectiveness and robustness of the proposed algorithm from the aspects of transmitters, sensors, measurement noise, and complexity. With the improved gated convolution and parallel processing of multiple tasks, the proposed MT-GCNN framework achieves more accurate localization than other DL-based algorithms in urban scenarios.

## Figures and Tables

**Table 1:** The comparison of grid-classification accuracy and mean positioning error with different algorithms when M=1 and N=4.

Algorithms	Grid-Classification Accuracy α (%)	Mean Positioning Error ε (m)
MT-GCNN	98.25	5.73
GCNN	96.54	59.35
CellinDeep	93.00	59.95
MLP	93.21	62.75

**Table 2:** The comparison of the mathematical complexity and testing time.

Algorithms	Time Complexity	Space Complexity	Testing Time (s)
MT-GCNN	$\mathrm{Time}∼O\left(9.86\times 10^{6}\right)$	$\mathrm{Space}∼O\left(8.38\times 10^{5}\right)$	9.3447
GCNN	$\mathrm{Time}∼O\left(7.89\times 10^{6}\right)$	$\mathrm{Space}∼O\left(6.71\times 10^{5}\right)$	8.8990
MLP	$\mathrm{Time}∼O\left(2.74\times 10^{5}\right)$	$\mathrm{Space}∼O\left(2.74\times 10^{5}\right)$	2.1693

## Data Availability

Data are contained within the article.

## Abbreviations

The following abbreviations are used in this manuscript:

AOA	Angle of Arrival
CNN	Convolutional Neural Network
CS	Compressive Sensing
DL	Deep Learning
DNN	Deep Neural Network
DPM	Dominant Path Model
FLOPs	Floating Point Operations
GCNN	Gated Convolutional Neural Network
GPU	Graphics Processing Unit
IoT	Internet of Things
LOS	Line of Sight
LSTM	Long Short-Term Memory
MLP	Multilayer Perceptron
MSE	Mean Square Error
MT-GCNN	Multi-Task Gated Convolutional Neural Network
MTL	Multi-Task Learning
NLOS	Non Line of Sight
RAM	Random Access Memory
RNN	Recurrent Neural Network
RSS	Received Signal Strength
TDOA	Time Difference of Arrival
TOA	Time of Arrival
WSN	Wireless Sensor Network
